# Significant reduction in errors associated with nonbonded contacts in protein crystal structures: automated all-atom refinement with *PrimeX*

**DOI:** 10.1107/S0907444912017453

**Published:** 2012-07-17

**Authors:** Jeffrey A. Bell, Kenneth L. Ho, Ramy Farid

**Affiliations:** a Schrödinger, 120 West 45th Street, 17th Floor, New York, NY 10036, USA

**Keywords:** H atoms, van der Waals radii, restraints, nonbonded contacts, clashes, molecular geometry, model quality, force fields, refinement, riding H atoms, electrostatics, hydrogen bonds

## Abstract

All-atom models are essential for many applications in molecular modeling and computational chemistry. Non­bonded atomic contacts much closer than the sum of the van der Waals radii of the two atoms (clashes) are commonly observed in such models derived from protein crystal structures. A set of 94 recently deposited protein structures in the resolution range 1.5–2.8 Å were analyzed for clashes by the addition of all H atoms to the models followed by optimization and energy minimization of the positions of just these H atoms. The results were compared with the same set of structures after automated all-atom refinement with *PrimeX* and with nonbonded contacts in protein crystal structures at a resolution equal to or better than 0.9 Å. The additional *PrimeX* refinement produced structures with reasonable summary geometric statistics and similar *R*_free_ values to the original structures. The frequency of clashes at less than 0.8 times the sum of van der Waals radii was reduced over fourfold compared with that found in the original structures, to a level approaching that found in the ultrahigh-resolution structures. Moreover, severe clashes at less than or equal to 0.7 times the sum of atomic radii were reduced 15-­fold. All-atom refinement with *PrimeX* produced improved crystal structure models with respect to nonbonded contacts and yielded changes in structural details that dramatically impacted on the interpretation of some protein–ligand interactions.

## Abstract 2

All-atom models derived from moderate-resolution protein crystal structures contain a high frequency of close nonbonded contacts, independent of the major refinement program used for structure determination. All-atom refinement with *PrimeX* corrects many of these problematic interactions, producing models that are better suited for use in computational chemistry and related applications.

## 1. Introduction   

The majority of protein crystal structures are solved in the resolution range 1.7–2.8 Å, a resolution range in which the diffraction experiment does not present sufficient information to accurately place individual atoms without additional chemical information. Electron-density peaks specifically for H atoms are not observed in this resolution range owing to a low signal-to-noise ratio. Therefore, H atoms are usually not explicitly included in molecular models of protein crystal structures. A molecular model without explicit coordinates for H atoms is denoted as an united-atom model, in contrast to an all-atom model. United-atom models are frequently insufficient for molecular modeling and computational chemistry applications (such as structure-based virtual screening or lead optimization). How is the gap bridged between current best crystallographic practices and the requirements of these other disciplines for all-atom structures that include hydrogen coordinates?

A brief history of the use of H atoms and chemical restraints in protein crystal structure refinement is useful before answering this question. Jensen and coworkers (Watenpaugh *et al.*, 1973 ▶) first demonstrated that moderate-resolution protein crystal structures could benefit from the reciprocal-space refinement techniques developed for use with crystal structures of small molecules at atomic resolution. They recognized the necessity of using additional chemical information combined with reciprocal-space refinement to accurately determine atomic positions in this situation.

A complete system of geometric restraints was devised for the first widely used protein reciprocal-space refinement program, *PROLSQ* (Konnert, 1976 ▶; Konnert & Hendrickson, 1980 ▶; Hendrickson, 1985 ▶). H atoms were not explicitly considered in this system.

The introduction of simulated-annealing refinement led to the widespread adoption of the program *X-PLOR* (Brünger, 1992 ▶). This program featured geometric restraints based on the CHARMM force field (Brünger *et al.*, 1986 ▶, 1989 ▶; Brünger & Karplus, 1988 ▶). Originally, use of this force field required an all-atom model. CHARMM-based restraints evolved in a way that removed the requirements for hydrogen coordinates. This change was associated with an alteration in the representation of nonbonded contacts from a Lennard–Jones potential to a much simpler repulsive function and the elimination of the use of electrostatic potentials. These modifications were partially motivated by electrostatic artifacts that were introduced into the structural results owing to the lack of an implicit solvent model. In addition, the long time required for computation of the complete set of nonbonded interactions was a significant impediment to the refinement of large crystal structures (Nilges *et al.*, 1988 ▶; Weis *et al.*, 1990 ▶). By the time that *X-­PLOR* was superseded by the program *CNS* (Brünger *et al.*, 1998 ▶), any requirement for explicit H-atom coordinates for protein crystallographic refinement had been eliminated. However, the capability to apply an electrostatic model and more complete nonbonded interactions in an all-atom model remained an essential part of *CNS* for the determination of structures from NMR data (Linge *et al.*, 2003 ▶).

Engh & Huber (1991 ▶) brought important additional information to the definition of the geometry for protein crystal structures. Their survey of bond lengths and angles observed in small peptide crystal structures at high resolution has been uniformly adopted as a standard against which protein crystal structure models are judged. It has also become the basis for the restraint system in all of the major refinement programs.

Recent developments indicate an interest among crystallographers in the application of more complex descriptions of molecular geometry in refinement to aid in producing better models. The refinement programs *REFMAC* (Murshudov *et al.*, 2011 ▶) and *PHENIX* (Afonine *et al.*, 2005 ▶) may be employed with ‘riding H atoms’, even though the ultimate result to be deposited is a united-atom model. (Riding H atoms are those H atoms whose positions can be determined unambiguously from the positions of the non-H atoms; for example, the H atom attached to the O^γ^ of a serine residue is not a riding H atom since its position depends on the torsion angle of the C^β^—O^γ^ bond, while the H atom on the C^α^ atom of an amino acid is a riding H atom, since all torsion angles affecting its position are determined by non-H atom coordinates.) The advantages of a restraint scheme in which geometric target values for a residue depend on the torsion-angle conformation of the residue backbone have recently been demonstrated (Tronrud *et al.*, 2010 ▶). Brunger and coworkers (Fenn *et al.*, 2010 ▶, 2011 ▶; Schnieders *et al.*, 2011 ▶) have combined the all-atom force field AMOEBA with a new refinement scheme and have described the advantages of a more complex molecular description that includes the calculation of electrostatic interactions between protein atoms. Additional recent innovations in the use of geometric information in refinement include the use of deformable elastic network refinement (Schröder *et al.*, 2010 ▶), hydropathic force-field terms (Koparde *et al.*, 2011 ▶) and jelly-body restraints (Murshudov *et al.*, 2011 ▶).

Structure-validation tools for protein geometry, partially based on the Engh & Huber standard, are available in several widely used computer programs, most notably *PROCHECK* (Laskowski *et al.*, 1993 ▶), *WHAT_CHECK* (Hooft, Vriend *et al.*, 1996 ▶), *NUCheck* (Feng *et al.*, 1998 ▶) and *SFCHECK* (Vaguine *et al.*, 1999 ▶). These programs address close non­bonded contacts largely from a united-atom perspective. More recently, the structure-validation programs *Reduce* and *MolProbity* (Davis *et al.*, 2007 ▶; Chen, Arendall *et al.*, 2010 ▶) have become important and popular additions to the toolkit of protein crystallographers. They are based on the concept that better judgments can be made as to the correct positioning of certain groups in the model after the addition of H atoms to a united-atom protein crystal structure and after observing their interactions. Within their software system, interpenetration of van der Waals molecular surfaces by 0.4 Å or more constitutes a clash. The authors flatly state that

Such large overlaps cannot occur in the actual molecule, but mean that at least one of the two atoms is modeled incorrectly

(Chen, Arendall *et al.*, 2010 ▶).

At this point, the question of the source of all-atom models needed for computational work can be addressed more clearly. Currently, such all-atom models are produced by adding H atoms to the united-atom models produced by crystallography. For water molecules and for protein H atoms whose position is subject to some degree of freedom, *i.e.* non-riding H atoms, either a force-field-dependent or a rule-based method is employed to determine the positions of these H atoms in order to avoid close nonbonded contacts and to form hydrogen bonds as appropriate. Nevertheless, when H atoms are added in this way to a very large majority of protein crystal structures deposited in the Protein Data Bank (Berman *et al.*, 2000 ▶), multiple close nonbonded contacts between atoms are observed. One goal of this work is to document this observation and to try to understand why such interactions occur, the recent focus on protein structure validation with H atoms present notwithstanding.

The usual remedy in computational chemistry to these high-energy close contacts is to minimize the coordinates of the all-atom model against a force field, with non-H atoms restrained to their positions in the crystallography-derived model so that they do not deviate too far from their experimentally determined positions. This solution is less than ideal, because the method produces no feedback as to whether the all-atom model is still consistent with the experimental data. In other words, one does not know how far is too far. This procedure could be especially dangerous if the original clashes were caused by atoms that were significantly misplaced.

The refinement program *PrimeX* was implemented partially in response to these issues. It applies well established methods of protein crystal structure refinement (Bell *et al.*, 2012 ▶) combined with the all-atom OPLS force field (Jorgensen *et al.*, 1996 ▶; Kaminski *et al.*, 2001 ▶; Banks *et al.*, 2005 ▶) for geometric restraints. Aside from the presence or absence of H atoms in the model, these OPLS-based restraints differ in two specific respects from what have become the traditional restraint systems: (i) a Lennard–Jones description of both the attractive and repulsive components of van der Waals interactions replaces the simpler repulsive term of most Engh and Huber-based restraints and (ii) electrostatic interactions are treated, including a Surface Generalized Born model to account for implicit solvent effects (Ghosh *et al.*, 1998 ▶; Gallicchio *et al.*, 2002 ▶; Zhu *et al.*, 2007 ▶; Li, Abel *et al.*, 2011 ▶). The net effect of these differences is very significant. In a simpler restraint system, the bond-length targets are each a function of a single parameter according to the atom types involved in the bond. A similar situation occurs for bond angles. However, the bond-length and bond-angle targets specified by OPLS are a function of several parameters that can all affect a single bond length or bond angle. In other words, the restraint target for a particular bond length (or angle) is contingent on the local environment of the atoms involved. Touw & Vriend (2010 ▶) have shown that at least one type of protein bond angle is a complex function of the local environment and is not well described by a single Engh & Huber (1991 ▶) target angle. The target geometric values in the well characterized restraint system of Karplus and coworkers depend on the local backbone conformation of the protein (Tronrud *et al.*, 2010 ▶). That any particular force field can reproduce all such dependencies remains to be demonstrated, but potentially a force-field-based restraint system can more effectively adapt to local environments than current protein crystallography restraint systems.

Refinement of protein crystal structures with an all-atom model and a complete force field does much more than avoid errors whose remediation may seriously degrade the accuracy of the coordinates. The more detailed accounting for non­bonded interactions within the protein used in *PrimeX* can also produce a direct positive effect during refinement. While even small changes in the structure near a ligand-binding site can be critical for structure-based drug discovery, examples are presented to show how refinement with an all-atom model can result in large coordinate improvements at such sites.

## 2. Methods   

### 2.1. Data-set selection   

The members of the moderate-resolution protein data set used in this study were selected from the Protein Data Bank (Berman *et al.*, 2000 ▶). Candidate structures were limited to those deposited in 2010 to ensure that refinement followed modern practices and that sufficient time had passed for structures to be withdrawn if found to contain gross errors. Reflection data were required to have been deposited with the coordinates. Each entry was restricted to contain one or more protein chains but no DNA or RNA. The reported *R*_free_ values were limited to 0.28 or lower. Structures were required to have a high-resolution limit between 1.5 and 2.8 Å. The molecular mass of protein within the asymmetric unit was limited to be between 10 and 300 kDa. Proteins with homologous sequences were removed at 30% identity.

In addition, the *PrimeX*-calculated *R*_free_ was required to exceed the *R* value by at least 0.008. This requirement ensured that the test set deposited in the PDB was likely to be the one that was actually used in the refinement of the deposited coordinates. (The *R*_free_ calculated using the deposited test set was found to actually be lower than the calculated *R* factor in several cases, strongly indicating that the deposited test set was not used in the final refinement of the deposited coordinates.) Others have made similar observations about test-set entries in the PDB (Joosten *et al.*, 2009 ▶; Afonine *et al.*, 2010 ▶). A meaningful comparison of *R*_free_ values was not possible without using a single consistent test set.

The high-resolution reference data set used in this study was selected from the PDB with the following restrictions: (i) the deposition of diffraction data was required, (ii) coordinate sets were selected from entries containing protein but no DNA or RNA and with a high-resolution limit of 0.9 Å or better, (iii) proteins with homologous sequences were removed at 30% identity and (iv) only proteins refined with coordinates for H atoms were included in this set.

### 2.2. *PrimeX* crystallographic calculations and refinement   

The key features of *PrimeX* crystal structure refinement have been described in some detail elsewhere (Bell *et al.*, 2012 ▶). Only details relevant to this work are described below.

#### 2.2.1. Restrained reciprocal-space minimization   

Reciprocal-space coordinate minimization is applied in *PrimeX* with a maximum-likelihood target, using the formulation of Pannu & Read (1996 ▶). The *PrimeX* implementation follows the general concepts developed by Brünger and coworkers (Brünger, 1989 ▶; Brünger *et al.*, 1998 ▶). A maximum-likelihood target has been shown to improve the convergence of refinement and to reduce the effects of model bias (Murshudov *et al.*, 1997 ▶).

OPLS 2005 (Jorgensen *et al.*, 1996 ▶; Kaminski *et al.*, 2001 ▶; Banks *et al.*, 2005 ▶) is a general-purpose force field for modeling proteins, nucleic acids and small molecules. *PrimeX* applies this consistent molecular description as geometric restraints during the refinement of the atomic positions for all molecular components of large biological crystal structures. Restrained isotropic *B*-factor refinement is applied in *PrimeX* using an approach similar to that used in the program *CNS* (Brünger *et al.*, 1989 ▶).

Because of its dependence on the OPLS force field, *PrimeX* operates on all-atom models at all stages of refinement. H-­atom coordinates do not participate in crystallographic calculations and are not influenced directly by the diffraction data. The advantage of this approach is that the underdetermined nature of the crystallographic refinement calculation is not made worse by the many more parameters for H-­atom coordinates and *B* factors. H-atom positions are a function of the force field acting on all atoms, while the positions of non-H atoms are refined under the joint influence of crystallographic and force-field gradients. Thus, the H-atom coordinates are not biased towards the centre of mass of the electron-density distribution, as may occur in some forms of all-atom refinement (Coulson & Thomas, 1971 ▶).

For H atoms bonded to more electronegative atoms, electrostatic forces are a major determinant of nonbonded interactions and they must be evaluated during the calculation of geometric gradients in the refinement. Thus, *PrimeX* employs the complete molecular-mechanics description of atomic interactions embodied in OPLS, including electrostatic terms (Jorgensen *et al.*, 1996 ▶; Kaminski *et al.*, 2001 ▶; Banks *et al.*, 2005 ▶). Also included in the current *PrimeX* calculations was an optional implicit solvation term (Ghosh *et al.*, 1998 ▶; Gallicchio *et al.*, 2002 ▶; Zhu *et al.*, 2007 ▶).

An overview of the OPLS force field, and a description of the details of the second-generation Surface Generalized Born model used to implicitly account for solvation effects, have been provided by Li, Abel *et al.* (2011 ▶). Both the electrostatic and solvation calculations employ residue-based cutoffs of 15 Å for long-range interactions between neutral residues, of 30 Å between charged residues and of 20 Å between mixed charged and neutral residues. Such approximations and model features should be considered in the context of a trade-off between computational time and rigorous calculations, as discussed by Moulinier *et al.* (2003 ▶), who pioneered the use of the Generalized Born approach in refinement, and by Fenn *et al.* (2011 ▶), who have advocated the use of an alternate electrostatic model in refinement. Refinement incorporating a complete electrostatic description has been shown to lead to lower *R*_free_ values compared with refinement excluding these interactions (Knight *et al.*, 2008 ▶; Fenn *et al.*, 2010 ▶, 2011 ▶; Schnieders *et al.*, 2011 ▶).

#### 2.2.2. Simulated-annealing refinement   

Simulated-annealing refinement within *PrimeX* is implemented through the general-purpose molecular-modeling package *IMPACT* (Banks *et al.*, 2005 ▶), employing concepts for simulated-annealing refinement validated in the program *CNS* (Adams *et al.*, 1997 ▶). *PrimeX* simulated annealing provides two alternative energy models for dynamic simulation refinement. In the complete energy model, all molecular-mechanics terms are evaluated during the simulation. In the approximate method, the electrostatic and implicit solvation terms are not evaluated, a method similar to that employed in *CNS* (Adams *et al.*, 1997 ▶). All calculations in this work involved the complete energy model.

#### 2.2.3. Electron-density map calculations   

Map calculations in *PrimeX* are based on the SIGMAA weighting scheme of Read (1986 ▶), a data treatment that has been shown to decrease the bias in electron-density maps.

#### 2.2.4. Hydrogen-bond network optimization   

An additional hydrogen-bond optimization tool in *PrimeX* analyzes the locations of hydrogen-bond donors and acceptors to define clusters of such sites that might be connected through hydrogen bonds. Within each cluster, hydrogen bonding is evaluated using a rule-based method to find an optimal combination of the variable components of these systems. The structural features adjusted during hydrogen-bond optimization are (i) alcoholic H-atom positions; (ii) sulfhydryl H-atom positions; (iii) phenolic H-atom positions; (iv) charge and tautomeric states of aspartic acid and glutamic acid side chains; (v) charge states, tautomeric states and orientation (flip) of histidine side chains; (vi) orientation (flip) of asparagine and glutamine side chains; and (vii) positions of H atoms in water molecules. The goal of this procedure is to minimize the energy of the system by maximizing the number of hydrogen bonds while avoiding close high-energy nonbonded inter­actions. This is accomplished by enumerating plausible orientations for each rotatable hydrogen and water molecule by identifying nearby hydrogen-bond donors and acceptors. Initial solutions for the overall local hydrogen-bond network are then generated by iteratively choosing the optimal state for each species in turn until convergence, starting from a variety of random starting conditions. These initial solutions are then recombined with each other and further optimized *via* simulated annealing. The best solution obtained overall is then chosen. The hydrogen-bond optimization tool in *PrimeX* performs essentially the same tasks as a number of other hydrogen-bond optimization tools such as *NETWORK* (Hooft, Sander *et al.*, 1996 ▶) and *Reduce* (Word *et al.*, 1999 ▶).

#### 2.2.5. The polish refinement workflow   

The all-atom structures produced by *PrimeX* refinement, as described in Table 4, were the result of application of the ‘polish’ workflow to the united-atom models obtained from the PDB without any human intervention during the refinement process. The refined coordinates that were the result of this process are archived at http://www.schrodinger.com/primex.

The purpose of the polish workflow is to produce the best all-atom model possible that is consistent with the diffraction data, starting with an already well refined crystal structure. The workflow applies reciprocal-space optimization of co­ordinates and thermal factors, simulated-annealing refinement and hydrogen-bond optimization in an automated manner as described below. It does not have as a purpose the remediation of more serious errors in crystal structure fitting such as the choice of the wrong side-chain rotamer, mis-identification of protein electron density as part of the solvent model or the rebuilding of misplaced side chains, which would require the application of additional fitting functions.

Bond orders are first assigned throughout the structure and H atoms are added. Initial analysis of the input structure provides basic crystallographic statistics for the structure using the bulk water correction in *PrimeX* (the flat model of Jiang & Brünger, 1994 ▶) and overall anisotropic scaling. A detailed analysis of close nonbonded contacts for the input structure is also provided.

As a next step, reciprocal-space minimization is applied at increasing weight on the X-ray terms (*w*_A_) in order to optimize this weight for subsequent refinement. The value selected corresponds to the weight employed when the minimum *R*_free_ value is observed. The weights selected for this set of proteins ranged between 0.25 and 1.73. Issues surrounding the selection of restraint weights when using an all-atom force field have been discussed by Fenn & Schnieders (2011 ▶). *B*-factor restraint weights (*w*_B_) are estimated from the high-resolution limit (*r*) of the diffraction data according to the equation 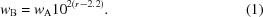
The functional form of this equation was chosen based on the known *B*-factor restraint-weight requirements in *PrimeX* at the bounds of the usual resolution range for refinement. For a low-resolution structure of 2.7 Å or worse, a weight of at least ten times *w*_A_ is required. For refinement at high resolution (better than 1.7 Å), a very low *B*-factor restraint weight (<0.1 times *w*_A_) is required. The values of the two constants in the equation were varied while observing the *R* factors from refinement in a broad resolution range. The current equation was observed to be as effective within *PrimeX* as stepwise optimization of *w*_B_ as described for *w*_A_ above and required much less computational time. Continued development of this method, such as the exploration of any effect of noncrystallographic symmetry restraints, will be reported in future work. Although this equation is the default method for assigning *B*-factor restraint weights in the polish workflow, stepwise optimization of this value is provided as an option. Also, minimization performed during weight optimization may optionally be applied towards progress in the refinement of the structure.

An initial optimization of hydrogen-bond orientation is applied and is followed by separate coordinate and *B*-factor reciprocal-space minimization steps. The model is then refined with a defined set of operations comprised of reciprocal-space coordinate minimization, hydrogen-bond optimization, reciprocal-space coordinate/*B*-factor minimization, simulated annealing and a final reciprocal-space coordinate/*B*-factor minimization. The optimization of X-ray and *B*-factor restraint weights as described above is repeated after this first refinement round. The same defined set of refinement procedures is then repeated twice more but without simulated annealing.

### 2.3. Direct generation of all-atom models from selected structures   

To generate the all-atom models described in Table 3, H atoms were added to united-atom models from the PDB and the positions of the H atoms were optimized using the hydrogen-bond network optimization function described above (§[Sec sec2.2.4]2.2.4) as implemented in the *Protein Preparation Wizard* (*Maestro* v.9.2; Schrödinger LLC). The positions of all H atoms were then also optimized through energy minimization against the OPLS force field, with the positions of all heavier atoms held fixed.

### 2.4. Structure-validation calculations   

#### 2.4.1. Clash detection and van der Waals radii   

The Rowland & Taylor (1996 ▶) compilation of van der Waals radii was employed in this study. It was based on an analysis of 28 403 structures in the Cambridge Structural Database (Allen, 2002 ▶). Their results agreed well with the frequently cited van der Waals radii derived by Bondi (1964 ▶) when the available solid-state structural data were not nearly so extensive. The largest difference between the two studies was that the radius of the H atom was determined to be 1.1 Å rather than 1.2 Å as in the older work.

As observed by Rowland & Taylor (1996 ▶), atoms may at times have nonbonded interactions somewhat less than the sum of their van der Waals radii. For the purposes of this work, a center-to-center distance of less than or equal to 0.8 times the sum of the van der Waals radii was defined as a ‘clash’. A reasonable conclusion from the selection of data presented by Rowland and Taylor is that interatomic distances of less than or equal to 0.7 times the sum of van der Waals radii are rare. Such interactions were denoted as ‘severe clashes’ in this work.

The current work focuses specifically on interactions among atoms within the proteins since this issue is the central concern for computational chemistry applications. Close interactions with water and solvent molecules will be the focus of future work. Clashes generated from symmetry considerations were not counted for observations on the moderate-resolution data set since they are not optimized by the version of the *Protein Preparation Wizard* used in this study.

Interatomic contacts were calculated after removing hydrogen bonds from consideration. Because of the lack of certainty regarding the positions of H atoms, all donor–acceptor atom pairs that could potentially be involved in a hydrogen bond were also excluded from the list of close contacts, even if an H atom was not found directly between them. This conservative approach avoided over-reporting as clashes any interactions that might actually be hydrogen bonds. Where alternate conformations were found, only the conformation with the higher occupancy was considered for the calculation of clashes.

The definition of a clash most commonly used in protein crystallography derives from the work of Jane Richardson, David Richardson and coworkers (Word *et al.*, 1999 ▶; Davis *et al.*, 2007 ▶; Chen, Arendall *et al.*, 2010 ▶). It is simply the overlap of two van der Waals surfaces by 0.4 Å or more. It is employed with an atomic radius of 1.00 Å for polar and aromatic H atoms and a radius of 1.17 Å for all other H atoms, resulting in clashes between two H atoms with the same radii at separations of 1.60 and 1.94 Å, respectively. Clashes between two H atoms in the current work occur at a separation of 0.8 times the sum of their van der Waals radii, *i.e.* 1.76 Å. For most other atoms the definition applied here is less strict than that applied by the Richardson group. The one exception is for O atoms, which have a smaller radius in the Richardson system, resulting in clashes between two O atoms at 2.40 Å separation compared with 2.53 Å in the current work. The Richardson atomic parameters were designed from various theoretical and practical considerations (Word *et al.*, 1999 ▶) to yield a system in which all observed clashes were exceptional. The approach in the current work was to use the values for atomic radii (Rowland & Taylor, 1996 ▶) unadjusted and to observe from ultrahigh-resolution protein structures how frequently clashes might reasonably be expected to occur owing to the local molecular environment.

#### 2.4.2. Calculation of summary geometry statistics   

Bond-length, bond-angle and torsion-angle statistics were calculated in *NUCheck* (Feng *et al.*, 1998 ▶). Side-chain group planarity deviation was calculated using the Protein Reports utility in *PrimeX*.

## 3. Results   

### 3.1. Ultrahigh-resolution structures   

Before exploring the close contacts (clashes and severe clashes as defined above) and structural geometry at moderate resolution, some perspective can be obtained on summary geometric statistics and the occurrence of clashes from ultra-high-resolution protein structures.

#### 3.1.1. Clashes and severe clashes   

Table 1 ▶ shows observations from 18 X-ray crystal structures with at least 0.9 Å resolution which were refined (by the authors of the respective structures) with H atoms present. (At this resolution hydrogen positions may have been guided by electron density, although electron density need not have been observed for all H atoms.) Over the entire set of structures, ‘clashes’ and ‘severe clashes’ were observed with a frequency of 1.5 and 0.6 occurrences per 100 residues, respectively.

All of the observed clashes were examined individually to determine their origin. The results are presented in Table 2 ▶. In 37% of the clashes a chemically implausible interaction was observed in which a hydroxyl or sulfhydryl H atom pointed directly at another H atom. The positions of these H atoms were not supported in any obvious way by the observed electron density. The most likely explanation is that these hydrogen positions were oversights in the model-building process. In addition, 21% of the close interactions identified occurred at positions where the heavy atoms to which the H atoms were attached did not fit the electron density well and appeared to be incorrectly positioned. Clashes were included in this category only if the electron-density map provided reasonable doubt as to the correctness of the structure and suggested a more attractive alternate position. A further 12% of close contacts could be removed by flipping or changing the tautomer of an asparagine, glutamine or histidine side chain.

Ultimately, only 30% of the observed clashes withstood critical examination and avoided being included in Table 2 ▶. In Table 1 ▶, the ‘corrected’ columns offer a better estimate of the close contacts actually present in the structures. Thus, the frequency of bona fide ‘clashes’ and ‘severe clashes’ was 0.6 and 0.03 per 100 residues, respectively. Note that the second value is based on just a single observation (one severe clash in 2xu3).

#### 3.1.2. Summary geometry statistics   

The average r.m.s. deviations (r.m.s.d.s) of bond lengths and angles with respect to the Engh & Huber (1991 ▶) standard were 0.021 Å and 2.5°, respectively (Table 1 ▶). The significance of the former number might be questioned since at this resolution the refined bond lengths are likely to reflect the effects of restraints. However, atomic positions are observed accurately enough at this resolution such that bond angles can be precisely determined from the crystallographic results. Thus, the latter value may be significant. However, two observations must be considered in interpreting this bond-angle value. Firstly, the program *SHELX* (Sheldrick & Schneider, 1997 ▶) may apply bond-angle restraints (as 1,3-atom distance restraints) during refinement, although published information rarely allows one to deduce the effect of these possible restraints on bond angles. Normally, one might expect the net effect of restraints would be to narrow the distribution of values observed. At the same time, a critical observation is that high-resolution protein crystal structures very frequently have r.m.s. *Z* scores (Spronk *et al.*, 2004 ▶) greater than 1, *i.e.* the standard deviation of bond angles for these structures are greater than what would be predicted from the work of Engh & Huber (1991 ▶). This observation (Joosten *et al.*, 2009 ▶) is generally interpreted to mean that the bond angles are too widely distributed in very high resolution structures, possibly because these restraints are faulty owing to variations in bond lengths at high resolution. The possibility that the Engh and Huber parameters predict too narrow a distribution owing to biases in the small-molecule structures from which the parameters were derived is generally discounted.

The average r.m.s.d. from planarity for side-chain groups was 0.011 Å and the average standard deviation of the ω angle was 6.4°. For similar reasons, these two values may be considered to be reference points for the geometry of models at moderate resolution.

### 3.2. Moderate-resolution crystal structures as deposited   

94 crystal structures with a broad range of sizes from several different refinement programs were examined (Table 3 ▶). Their most important common characteristics were recent deposition in the PDB and falling into the most highly populated resolution range typical for protein crystal structures (see §[Sec sec2]2 for further details on the selection of this data set).

#### 3.2.1. Clashes and severe clashes   

The occurrence of close contacts was enumerated after addition of H atoms and after careful optimization of H-atom positions without changing the coordinates of any non-H atoms. Table 3 ▶ shows the frequency of clashes and severe clashes for each protein. Their rates of occurrence per 100 residues were observed in a very broad range from 20.4 (3lpf) to 0.0 (three instances) and from 2.9 (3lpf) to 0.0 (35 instances) for clashes and severe clashes, respectively. Overall, clashes in the moderate-resolution structure set were observed at a frequency of 4.0 per 100 residues, over six times the rate of bona fide clashes in the ultrahigh-resolution set. Severe clashes were observed at a frequency of 0.5 per 100 residues, compared with 0.03 for bona fide severe clashes for the reference ultrahigh-resolution data set (Table 1 ▶).

#### 3.2.2. Summary geometry statistics   

As shown in Table 3 ▶, the bond-length r.m.s.d.s for the set of proteins varied over a wide range, from 0.004 Å for 3phe to 0.031 Å for 3lje, with an average of 0.014 Å. Bond-angle r.m.s.d.s varied from a minimum of 0.6° (3ni0) to a maximum of 2.5° (3nof), with an average of 1.4°. The average r.m.s.d. for side-chain group planarity was 0.005 Å and the average peptide torsion-angle standard deviation was 5.1°. A more detailed examination and comparison of these summary statistics follows in §[Sec sec3.3.2]3.3.2.

### 3.3. All-atom refinement with *PrimeX*   

#### 3.3.1. Clashes and severe clashes   

The additional all-atom refinement in *PrimeX* applied to the moderate-resolution data set produced the structures characterized in Table 4 ▶. The frequency of regular clashes overall was 0.9 per 100 residues, which is well below the frequency originally observed for the ultrahigh-resolution set (1.5 per 100 residues; Table 1 ▶), but somewhat higher than the corrected value of 0.6 per 100 residues. The frequency of clashes overall was decreased more than fourfold from all-atom models derived from the coordinates as originally deposited. The frequency of severe clashes overall was 0.03 per 100 residues, the same value as obtained for the corrected ultrahigh-resolution structures (Table 1 ▶) and 17-­fold lower than the frequency in the otherwise remediated moderate-resolution structures (Table 3 ▶). Seven of the 94 structures had neither type of clashes after all-atom refinement. All structures without clashes were solved at 2.2 Å resolution or better. 39 of the 94 structures had both no severe clashes and a lower frequency of clashes than the corrected ultrahigh-resolution structures.

Each of the residual clashes in the *PrimeX*-refined set was inspected with reference to a 2*F*_o_ − *F*_c_ composite OMIT map. Clear evidence of a better alternate interpretation of the electron density was present for 14% of the close contacts, owing to either large problems with the main-chain fit or to the need for a substantially different side-chain rotamer. Subtracting the number of clashes attributable to these issues from the total clashes provided an estimate of the frequency of bona fide regular clashes as 0.7 clashes per 100 residues, approaching the corrected frequency found in the ultrahigh-resolution structure set (0.6 clashes per 100 residues). Within this structural survey, many situations were observed to be ambiguous and were not counted. Thus, the level of clashes owing to model errors might actually have been somewhat higher. A very time-intensive comprehensive re-refinement of the structures would be required to confirm this suspicion, which is beyond the scope of the present work.

Clash frequencies derived from Tables 1 ▶, 3 ▶ and 4 ▶ are compared in Table 5 ▶, as well as with respect to the various refinement programs. Unfortunately, refinement results from *BUSTER* (Bricogne *et al.*, 2011 ▶) were found to be relatively rare and only two instances were found in our data set. However, the statistics for these two proteins suggest improved results from this program regarding close contacts. None of the other programs even approached the values of 0.6–0.7 clashes per 100 residues that might be considered a reasonable target considering the results above.

#### 3.3.2. Summary molecular geometry and refinement statistics   

Table 5 ▶ also provides a summary of the measures of molecular geometry over the three data sets in this study. The average bond-length r.m.s.d. for the *PrimeX*-refined proteins was 0.019 Å, compared with 0.015 Å for the original data set. The r.m.s. *Z* scores (Spronk *et al.*, 2004 ▶) for bond lengths changed from an average value of 0.56 (0.15–1.27) as deposited to an average value of 0.89 (0.65–1.24) after *PrimeX* refinement. The ultrahigh-resolution set had a bond-length r.m.s.d. of 0.021 Å and a mean bond-length r.m.s. *Z* score of 0.87 (0.49–1.23), values that are very similar to those of the *PrimeX*-refined structures. Individual bond-length r.m.s. *Z* scores are available as Supplementary Material^1^.

Table 5 ▶ also allows comparison among the four programs originally used to refine the moderate-resolution data set. *PHENIX* and *CNS* clearly restrained bond lengths more tightly than did *PrimeX*. The bond-length r.m.s.d. for the *REFMAC*-refined set was not very different from the *PrimeX*-refined set.

The average bond-angle r.m.s.d. for *PrimeX* was 2.2°, which is somewhat larger than the average value of 1.4° over the original data set. The r.m.s. *Z* scores for bond angles changed from an average value of 0.71 (0.37–1.25) as deposited to an average value of 1.17 (1.00–1.49) after *PrimeX* refinement. The average bond-angle r.m.s.d. in the ultrahigh-resolution set was 2.5°, which is greater than that produced by any of the other refinement programs, but closest to the value for *PrimeX*. The r.m.s. *Z* score for bond angles in the ultrahigh-resolution set was 1.12 (0.79–1.46), which is also similar to that of the *PrimeX*-refined structures. Individual bond-angle r.m.s. *Z* scores are available in the Supplementary Material^1^.

The average side-chain group planarity r.m.s.d. for *PrimeX* was 0.006 Å, which is a fairly typical value for this quantity among the refinement programs. Side-chain planarity deviations were all small and not very different between these two data sets, nor did they differ much by refinement program (Table 5 ▶).

The average ω-angle standard deviation for *PrimeX*, 7.1°, was larger than for any of the refinement programs used to produce the original moderate-resolution data set, but com­pared well with the value of 6.4° obtained for the ultrahigh-resolution set (Table 5 ▶). The values among all the refinement programs could be described as a range of values from 4.8° to 5.7°, with two outliers near 2° for *BUSTER* and *CNS*/*CNX*. The number of examples of *BUSTER*-refined proteins was too small to draw a conclusion. However, *CNS* and *CNX* clearly often restrain the ω angle very tightly. This issue was originally observed by Priestle (2003 ▶). Note that the overall average for the ω angles did not represent the situation well, since two of the 12 structures in the *CNS*/*CNX* subgroup had standard deviations in the normal range (Table 3 ▶). These two uncharacteristic *CNS*/*CNX* structures indicate that at least a few users of *CNS*/*CNX* have taken steps to loosen these peptide-bond planarity restraints. Ten of the 12 members of the *CNS*/*CNX* subgroup had standard deviations for ω of 1.5° or less, implying flattened peptide bonds throughout these crystal structures. The ω-angle standard deviation did not vary much among the other refinement programs. The average value for *REFMAC* did not stand out as the one from *CNS*/*CNX* does. At the same time, some users of *REFMAC* did very tightly restrain peptide bonds. The lowest standard deviation for ω was not from among the *CNS*/*CNX*-refined structures, but instead was produced by *REFMAC* (3pgj; 0.9°).

Changes in overall structure quality owing to *PrimeX* refinement, as judged by the Ramachandran *Z* scores (Spronk *et al.*, 2004 ▶), were generally small. Only one change was noted as significant by the program *WHAT_CHECK*. This change was from an original value of −3.16 for the protein 3nl6 as originally deposited to a value of −2.11 after *PrimeX* refinement. The mean Ramachandran *Z* score changed from −0.37 (range −3.16 to +3.69) as originally deposited to −0.51 (range −2.56 to +3.05) after *PrimeX* refinement. Individual Ramachandran *Z* scores before and after *PrimeX* refinement are shown in the Supplementary Material^1^.

The average *R*_free_ value over the moderate-resolution set was the same with or without the additional *PrimeX* all-atom refinement (0.243 *versus* 0.242; Tables 2 ▶ and 3 ▶). The average for all working *R* values was somewhat lower for the *PrimeX*-refined structures (0.193) *versus* the average from the original structures (0.207).

### 3.4. Additional benefits from all-atom refinement   

The advantages of all-atom refinement of structures at moderate resolution extend well beyond the prevention and remediation of clashes. A few examples from the *PrimeX* refinements in this study illustrate how a detailed description of nonbonded contacts influenced and improved the results of refinement.

#### 3.4.1. Repositioning of a methionine methyl group   

**Figure 1:**
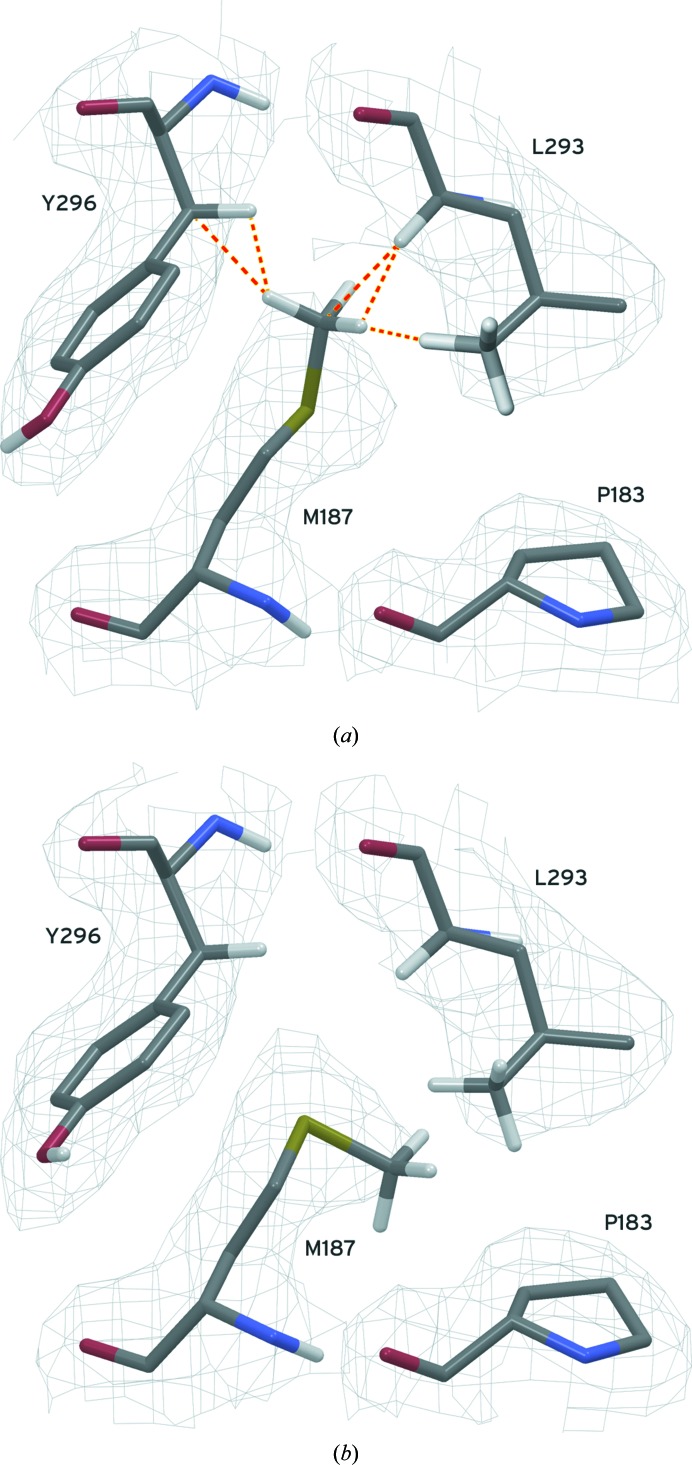
MetC187 in PDB entry 3phe is shown in (*a*) as originally refined in *CNX*, with only a selection of H atoms added for clarity. Clashes between the terminal methyl group of the methionine and two other residues are shown as orange dashed lines. These close interactions were tolerated during the original refinement as a united-atom model. In (*b*) the location of the methyl group after *PrimeX* refinement is shown, where no clashes involving the methyl group were observed. The electron-density grid for this region is contoured at 1.0σ from a 2*F*_o_ − *F*_c_ composite OMIT map.

Fig. 1 ▶ provides an example in which all-atom refinement used in *PrimeX* led to a significant improvement in the structural model. In PDB entry 3phe, clashes of the C^∊^ and associated H atoms of MetC187 with atoms from LeuC293 and TyrC296 suggest that at least one of these residues is in the wrong position. *PrimeX* refinement using the ‘polish’ workflow moved the methyl group as shown in Fig. 1 ▶ without manual intervention. The 2*F*_o_ − *F*_c_ electron-density map as shown did not give any clear indication of the correct position for this methyl group. However, the position as deposited was un­favourable and unlikely to be correct as judged from the observed clashes. The new position for the methionine methyl group relieved all close contacts and was confirmed by a small pair of negative and positive difference features in an *F*_o_ − *F*_c_ map (result not shown). The refinement program *CNX* did not correct this situation. A reasonable hypothesis for why it did not do so is that the interactions between the methionine methyl group and the other two residues, as represented through a united-atom model in *CNX*, were not unfavorable enough to cause a change in the positions of these atoms.

#### 3.4.2. Backbone change to relieve clash leads to additional ligand hydrogen bonds   

**Figure 2:**
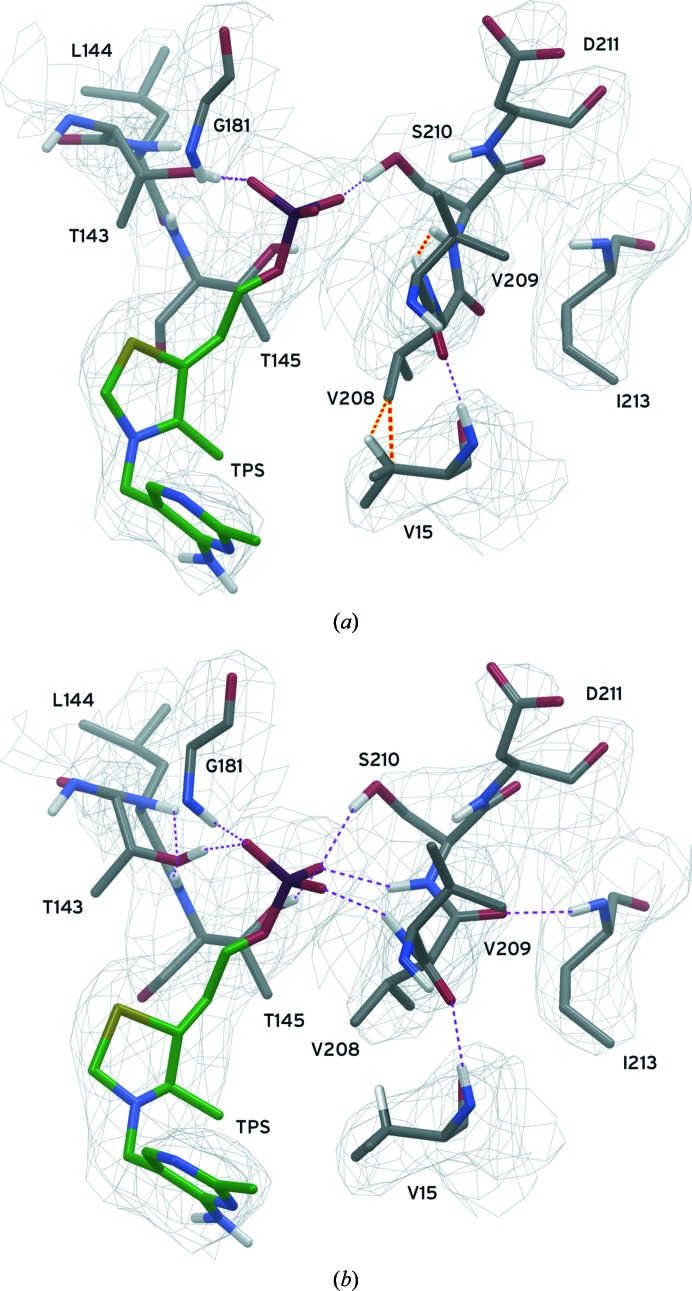
The region near ValC209 in PDB entry 3nl6 is shown in (*a*) as originally produced with *phenix.refine*. All H atoms were added to this model and their positions were minimized while holding non-H atoms in fixed positions, but only some of these H atoms are shown for clarity. The extremely close contacts between the H atom attached to C^β^ of ValC15 and atoms of the ValC209 side chain (orange dashed lines) distorted the bond angles around C^β^. The coordinates of residues C209 and C210 changed after *PrimeX* refinement as shown in (*b*), with a shift in the backbone conformation, relieving the close contacts and resulting in multiple additional hydrogen bonds to the ligand (purple dashed lines). The electron-density grid for this region is contoured at 1.0σ from a 2*F*_o_ − *F*_c_ composite OMIT map.

In PDB entry 3nl6, atoms in the side chain of ValC209 clash with the side chain of ValC15 (Fig. 2 ▶). In producing the all-atom model derived from this structure through energy minimization, these interactions were sufficiently repulsive that the bond angles around C^β^ of ValC15 were distorted rather than allowing atoms to overlap to such an extreme extent. The close contact was relieved during *PrimeX* refinement using the ‘polish’ workflow by motion of residues C209 and C210 away from residue C15 and towards the bound thiamine phosphate (TPS), as shown in Fig. 2 ▶. This side-chain motion occurred with a change in the conformation of the main chain for residue C209. This change in the backbone position and a few other more subtle atomic shifts provided multiple additional hydrogen-bond interactions between the protein and the TPS molecule, a difference that has potentially major implications for the understanding of TPS binding. This large structural change during refinement was probably related to the resolution of the strain of close contacts in the model, but electrostatic gradients or other influences during refinement could also play a role. Assuming that the program was used as intended and in the absence of any indication to the contrary (Paul *et al.*, 2010 ▶), *phenix.refine* seems to have tolerated these severe implied all-atom clashes during refinement.

#### 3.4.3. Refinement of two side-chain positions provides a new view of ADP binding   

**Figure 3:**
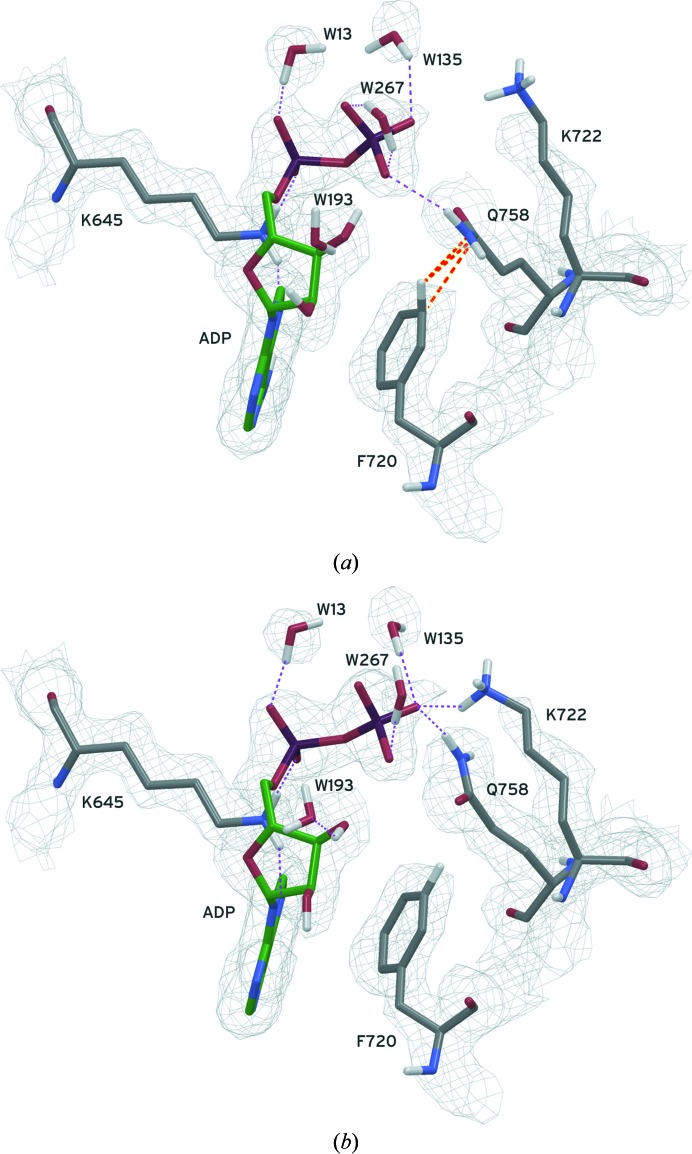
The region near ADP A811 in PDB entry 3pdt is shown in (*a*) as originally refined in *REFMAC*. All H atoms were added to this model and their positions were minimized while holding non-H atoms fixed, but only some of these H atoms are shown for clarity. Multiple clashes between the misplaced residue GlnA758 and PheA720 were apparently tolerated in the *REFMAC* refinement. The close contacts (orange dashed lines) were so severe that the energy minimization distorted the planarity of the aromatic system rather than allowing the higher energy interpenetration of atoms. The position of these two residues after refinement with *PrimeX* is shown in (*b*). In order to relieve the strain of the clash, the LysA722 side chain moved toward the ligand with a coordinated motion of the glutamine side chain into its correct position in strong electron density below the Lys residue. Hydrogen bonds are shown as purple dashed lines. The electron-density grid for this region is contoured at 1.0σ from a 2*F*_o_ − *F*_c_ composite OMIT map.

In its original position in PDB entry 3pdt, as refined in *REFMAC*, a clash occurred between GlnA758 and PheA720 in the all-atom structure (Fig. 3 ▶). The change in structure after *PrimeX* refinement using the ‘polish’ workflow is hypothesized to have occurred through the following chain of events. The movement of the GlnA758 side chain was first driven by relief of this clash. Concurrently, the LysA722 side chain was moved towards the phosphate group of the ADP molecule under the influence of both the force field and electron-density gradients, which also required the motion of the glutamine to avoid the formation of a clash with the lysine. Whatever the causes, the result was a large co­ordinated movement of the lysine and glutamine side chains which was dramatic both in terms of the extent of the motion of the glutamine side chain and in the difference in the key interactions observed for the binding of ADP to this protein.

The molecular model as originally deposited contains several side chains, including GlnA758, that are misfitted and thus this structure might be considered by some to be a poor candidate for automated refinement. In this alternate view of the situation shown in Fig. 3 ▶, residue GlnA758 is positioned outside of the anticipated radius of convergence for refinement. However, one conclusion is clear: *REFMAC* was tolerant of the implied clash as described above either because it was designed to behave so or because a decision by the users (Crawley *et al.*, 2011 ▶) caused *REFMAC* to behave in this way. *PrimeX* all-atom refinement is not tolerant of such inter­actions because of the highly unfavorable energetics calculated for such an interaction and it does not allow users to modify its behavior to tolerate such interactions without extraordinary efforts. Even when considered in this context, the ability of the automated *PrimeX* polish workflow to improve the model in the manner described in Fig. 3 ▶ is encouraging.

## 4. Discussion   

### 4.1. Summary geometry statistics for *PrimeX* and other refinement programs   

While this study was primarily focused on close nonbonded contacts and refinement using an all-atom model, other issues regarding molecular geometry were also of interest and might best be discussed first. Only moderate-resolution structures deposited and released in 2010 were used in this study to ensure that the results reflected current practices in protein crystallography, especially with respect to geometric restraints.

Use of the OPLS all-atom force field in *PrimeX* produced reasonable results with respect to summary geometry that were in line with other programs in terms of bond-length deviation and side-chain group planarity (Table 5 ▶). The results from the two other summary geometry descriptors monitored here deserve additional comment.

The average of the bond-angle r.m.s.d.s for *PrimeX* (2.2°) is greater than for any of the other programs that created the original moderate-resolution structure set (range 1.1–1.7°; Table 5 ▶). However, the observation from the ultrahigh-resolution data set of an average bond-angle r.m.s.d. of 2.5° (range 1.4–3.1°; Table 1 ▶) clearly suggests that this r.m.s.d. is reasonable.

Over-restraint of ω angles in *CNS*/*CNX* has been recognized for several years (Priestle, 2003 ▶). Considering the time that has passed since this publication, the number of structures from *BUSTER*, *CNS*/*CNX* and *REFMAC* observed with very low deviation of ω angles is hard to understand. While over-restraint is easy to recognize, the correct degree of variability is less easy to define. MacArthur & Thornton (1996 ▶) suggested from their study of proteins and small polypeptides that a standard deviation of 6° is appropriate.

Forcing bond angles or torsion angles toward idealized values does carry a risk. If an interaction such as a nonbonded repulsion has driven a particular torsion or bond angle away from the idealized value, restraining it to be closer to the idealized value must make that other interaction more un­favorable. Thus, the result of strictly enforcing these ideal values could be an increase in the number or severity of clashes.

That the same *R*_free_ was obtained with our force-field-based restraints as with Engh and Huber restraints suggests that these restraints are reasonably consistent with protein crystal structures. However, taken together, the decrease in the average *R* factor (*R*_work_), the relatively large r.m.s.d. for bond angles compared with the deposited structures and the somewhat larger standard deviation for the ω angle above the optimal value conceived by MacArthur & Thornton (1996 ▶) could be interpreted as evidence that the restraints employed may require further tuning to decrease the risk of overfitting. This consideration will be examined in future publications.

### 4.2. Advantages of all-atom refinement with *PrimeX*   

All-atom refinement of moderate-resolution protein crystal structures with *PrimeX* resulted in a more than fourfold decrease in the number of clashes and a 17-fold decrease in the number of severe clashes. This improvement in model quality was achieved without sacrificing the goodness of fit to the X-ray data as judged by the average *R*_free_ values. Importantly, these models also display good summary statistics, so that the protein models comply with reasonable molecular-geometry expectations.

All-atom refinement with a force field allowed *PrimeX* refinement to fix errors that other refinement programs missed and provided a more accurate picture of critical protein features such as protein–ligand interactions, as illustrated in Figs. 1 ▶, 2 ▶ and 3 ▶. Resolution of clashes during refinement can help to ‘push’ the structure into the correct conformation, producing potentially remarkably large changes in conformation. They may also serve the role of preventing the structure from entering nonproductive conformations that are otherwise allowed in a less restrictive all-atom model.

These results also contain an indication of the limits of usefulness of the polish workflow. Fully 85% of the structures that entered the workflow with 50 or more total clashes (Table 3 ▶) resulted in an increase in *R*_free_ (Tables 3 ▶ and 4 ▶). A large number of clashes is a warning sign that the structure may contain errors that could have negative consequences after the application of this refinement process.

### 4.3. Reducing clashes in deposited X-ray crystal structure models   

The refinement programs *REFMAC* and *phenix.refine* both have the capability to use riding H atoms during refinement. One might reasonably expect that the use of this feature would have a positive impact on the issue of clashes in all-atom models. Unfortunately, the extent to which the riding H atom option is actually employed in refinement is impossible to determine in many cases, even after consulting both the primary literature references and the PDB entry. The lack of definitive information on this issue makes it nearly impossible to determine from these experiments whether these programs are capable of reducing clashes to the levels deduced to be reasonable goals from the ultrahigh-resolution structures or from the *PrimeX*-refined structures. Comparing the frequency of clashes in Tables 3 ▶ and 4 ▶, one can only conclude that either (i) the riding H atom models and nonbonded contact restraints do not make as much difference as one might expect, (ii) the riding H atom option is very rarely used in these two programs or (iii) both are true. At the very least, one may safely conclude that some attribute of these programs or the way that they are being used must change before either of these programs can be considered to be part of the solution to this problem.

A role for *CNS* in curbing clashes is also currently available. *CNS* can be employed with a more complex energy model than is routinely used by crystallographers. As well as deploying an Engh and Huber-based restraint system, *CNS* is distributed with a force field that includes Lennard–Jones and electrostatic terms and that is regularly used for the determination of NMR structures (Linge *et al.*, 2003 ▶). This force field has been employed to produce some very high quality NMR structures (see, for example, Nozinovic *et al.*, 2010 ▶).

What else can be done to reduce the number of clashes in deposited structures? Perhaps the answer to this question resides in the standards for structure deposition in the PDB. A committee of the PDB is currently working on structure-validation tools for use associated with the deposition of coordinates (Read *et al.*, 2011 ▶). A reason for optimism is that the work of the Richardson group was included in the report of the committee. From the point of view of many users of protein structures, the deposition of all-atom models derived from protein crystal structures should be required. Clashes determined from an all-atom model should be, at the very least, measured and documented for all protein models that are deposited, just as other outliers to molecular-geometry standards are now listed in the entry header.

To achieve a higher standard for deposited protein structures, additional tools that are sensitive to close contacts could help. *PrimeX* can contribute to this goal, and the automated polish workflow presented here was designed to achieve this goal with the minimum of human intervention. However, the workflow was designed with the assumption that the crystal structure coordinates on which it would operate would be essentially free of errors in the main-chain tracing or side-chain rotamer selection. The prevalence of such errors in the data set examined here established the need for additional automated structure tools with the capability of making large changes in side-chain torsion angles or chain trace. Design of these workflows is in progress based on the tool set in the *PrimeX* refinement package (Bell *et al.*, 2012 ▶). Although similar automated workflows exist for *phenix.refine* (Afonine *et al.*, 2005 ▶) and indirectly for *REFMAC* (Murshudov *et al.*, 2011 ▶) through the program *SideAide* in the *PDB_REDO* pipeline (Joosten *et al.*, 2011 ▶), the frequency of clashes in structures refined by *phenix.refine* and *REFMAC* (Tables 3 ▶ and 5 ▶) raises the question whether these automated workflows can address the issue of all-atom clashes, no matter how capable and thorough these workflows are intended to be. While the program *MolProbity* (Davis *et al.*, 2007 ▶; Chen, Arendall *et al.*, 2010 ▶) is aimed at solving the right problem, these same results show that it is not being adequately employed to deal with the problem at hand.

The results presented here also highlighted a lack of attention to detail during structure determination in the ultrahigh-resolution protein data set. H atoms should not be placed in chemically impossible positions when, by all appearances, convincing electron density at those positions is lacking. In addition, clashes highlighted several clear errors in the coordinates of non-H atoms.

## 5. Conclusion   

This study documents the existence of numerous unnecessary close contacts, including many severe ones, implicit in united-atom models deposited in the PDB. Many of these close contacts can be readily removed, and doing so need not damage the agreement of the model with the observed X-ray diffraction data. Furthermore, attention to close contacts can bring to light errors in the placement of non-H atoms in protein crystal structure models. This latter point has also been made abundantly clear by over a decade of work by Jane Richardson, David Richardson and coworkers (Word *et al.*, 1999 ▶, Davis *et al.*, 2007 ▶; Chen, Arendall *et al.*, 2010 ▶).

Clashes are detrimental to advances in the various branches of science that depend on protein crystal structure models, such as protein design and drug discovery. Normally, scientists working in these areas are not in a position to evaluate the reliability of each protein crystal structure, nor are they able to judge whether the effects of remediation of crystal structures might result in different sorts of errors. If crystallographers, who are of course in the best position to do so, do not address these issues, then eventually other scientists will. The result will be that protein crystallographers will have less control over the form in which their experimental results are archived and deployed. The advent of remediated database alternatives to the PDB (Joosten *et al.*, 2009 ▶) is partially an outgrowth of this problem and an indication that this anticipated consequence is already becoming a reality.

Both the expected bond-length and bond-angle parameters of Engh & Huber (1991 ▶) and the parameterization of van der Waals radii by Rowland & Taylor (1996 ▶) are equally well grounded in high-resolution small-molecule crystallographic results. In protein crystallography the former geometric statistics are very strictly applied, while the latter receive much less attention. An understandable explanation for this contrast is the absence of H-atom coordinates in classic protein crystal structure models. However, if the requirements for the highest quality protein models possible are to be met, the consideration of nonbonded contacts in all-atom models must become more prominent.

## Supplementary Material

Supporting information file. DOI: 10.1107/S0907444912017453/rr5017sup1.pdf

## Figures and Tables

**Table 1:** Characteristics of ultrahigh-resolution protein structures

									‘Corrected’		
PDB code	Resolution ()	No. of residues	Bond-length r.m.s.d. ()	Bond-angle r.m.s.d. ()	Side-chain planarity r.m.s.d. ()	-Angle standard deviation ()	Clashes†	Severe clashes‡	Clashes†	Severe clashes‡	Reference
1byz	0.90	52	0.019	2.0	0.006	2.9	0	1	0	0	Priv *et al.* (1999 ▶)
1dy5	0.87	248	0.020	2.6	0.013	6.6	8	2	2	0	Esposito *et al.* (2000 ▶)
1i1w	0.89	303	0.041	2.9	0.016	6.4	1	2	0	0	Natesh *et al.* (2003 ▶)
1m40	0.85	263	0.014	2.6	0.009	6.1	1	0	0	0	Minasov *et al.* (2002 ▶)
1muw	0.86	386	0.016	2.5	0.010	8.2	18	3	7	0	Fenn *et al.* (2004 ▶)
1p9g	0.84	41	0.017	2.4	0.009	6.7	0	0	0	0	Xiang *et al.* (2004 ▶)
1ucs	0.62	64	0.014	2.2	0.010	5.3	0	1	0	0	Ko *et al.* (2003 ▶)
1vyr	0.90	364	0.017	2.3	0.012	5.8	0	0	0	0	Khan *et al.* (2004 ▶)
1yk4	0.69	52	0.022	2.7	0.011	5.7	2	3	0	0	Bnisch *et al.* (2005 ▶)
2b97	0.75	142	0.028	2.8	0.016	7.6	2	0	0	0	Hakanp *et al.* (2006 ▶)
2h5c	0.82	198	0.025	2.4	0.015	7.3	2	0	2	0	Fuhrmann *et al.* (2006 ▶)
2vb1	0.65	129	0.021	3.1	0.012	7.1	1	0	0	0	Wang *et al.* (2007 ▶)
2wur	0.90	236	0.034	3.3	0.016	6.6	10	5	7	0	Shinobu *et al.* (2010 ▶)
2xu3	0.90	220	0.011	1.4	0.007	5.7	1	1	1	1	Hardegger *et al.* (2011 ▶)
3a38	0.70	83	0.028	2.9	0.010	6.8	0	0	0	0	Takeda *et al.* (2010 ▶)
3g63	0.88	381	0.014	2.1	0.009	6.7	2	0	1	0	Liebschner *et al.* (2009 ▶)
3ip0	0.89	158	0.012	1.5	0.007	6.1	1	0	0	0	Blaszczyk *et al.* (2003 ▶)
3mi4	0.80	223	0.028	2.5	0.015	6.8	3	3	1	0	A. Brzuszkiewicz, M. Dauter Z. Dauter (unpublished work)
Total		3543					52	21	21	1	
Mean			0.021	2.5	0.011	6.4					
Count per 100 residues							1.5	0.6	0.6	0.03	

† A clash occurs when two atoms approach to within less than or equal to 0.8 times the sum of their van der Waals radii but greater than 0.7 times that sum.

‡ A severe clash occurs when two atoms approach to within less than or equal to 0.7 times the sum of their van der Waals radii.

**Table 2:** Classification of clashes and severe clashes requiring correction in Table1 ▶

Classification	No. of clashes and severe clashes
Hydroxyl (or sufhydryl) group: rotation around carbonoxygen (sulfur) bond relieves clash	27
Mistake in positioning of non-H atoms in the electron density	15
Flip of asparagine or glutamine residue relieves clash	4
Flip or alternate tautomer of histidine residue relieves clash	5

**Table 3:** Statistics for structures of moderate-resolution data in the PDB

PDB code	Resolution ()	No. of residues	Refinement program	Bond-length r.m.s.d. ()	Bond-angle r.m.s.d. ()	Side-chain planarity r.m.s.d. ()	-Angle standard deviation ()	*R* factor†	*R*_free_ †	Clashes‡	Severe clashes§	Reference
2x3k	2.50	1145	*REFMAC*	0.015	1.5	0.004	6.7	0.216	0.266	58	7	Schmelz (2010 ▶)
2xda	1.85	150	*REFMAC*	0.014	1.3	0.005	5.7	0.189	0.216	2	1	Paz *et al.* (2011 ▶)
2xn8	1.64	409	*REFMAC*	0.023	1.8	0.009	6.2	0.181	0.220	6	0	Ouellet *et al.* (2011 ▶)
2xpp	1.74	161	*REFMAC*	0.018	1.6	0.005	4.8	0.208	0.252	5	0	Diebold *et al.* (2010 ▶)
2xs6	2.09	166	*REFMAC*	0.007	1.0	0.002	4.9	0.219	0.258	4	0	¶
2xsn	2.68	1341	*BUSTER*	0.013	1.6	0.008	2.8	0.224	0.260	13	2	J. R. C. Muniz, C. D. O. Cooper, W. W. Yue, E. Krysztofinska, F. Vondelft, S. Knapp, O. Gileadi, C. H. Arrowsmith, A. M. Edwards, J. Weigelt, C. Bountra, K. L. Kavanagh U. Oppermann (unpublished work)
2xsq	1.72	179	*REFMAC*	0.009	1.2	0.004	5.4	0.173	0.191	0	1	¶
2xsw	1.90	666	*PHENIX*	0.011	1.3	0.004	5.6	0.184	0.209	8	0	¶
2xsx	1.70	869	*REFMAC*	0.015	1.5	0.007	5.9	0.177	0.207	11	3	M. Vollmar, E. Krysztofinska, A. Chaikuad, T. Krojer, R. Cocking, F. Vondelft, C. Bountra, C. H. Arrowsmith, J. Weigelt, A. Edwards, W. W. Yue U. Oppermann (unpublished work)
2xu7	1.90	752	*REFMAC*	0.023	1.8	0.008	7.1	0.211	0.245	14	1	Lejon *et al.* (2011 ▶)
2xul	2.20	645	*REFMAC*	0.029	2.0	0.005	6.5	0.196	0.235	20	1	Fokina *et al.* (2010 ▶)
2xvs	1.80	166	*REFMAC*	0.016	1.5	0.007	5.8	0.200	0.249	0	0	Adams *et al.* (2012 ▶)
2xvv	2.40	582	*CNS*	0.007	1.2	0.005	1.0	0.211	0.252	35	1	Ryan *et al.* (2011 ▶)
2xxj	1.96	1239	*PHENIX*	0.007	1.0	0.003	9.0	0.190	0.237	27	3	J. Tickle, E. De Mendoza Barbera F. M. D. Vellieux (unpublished work)
3acw	1.63	284	*REFMAC*	0.007	1.5	0.006	4.9	0.228	0.252	5	0	Lin *et al.* (2010 ▶)
3aey	1.92	698	*CNS*	0.005	1.2	0.004	1.2	0.192	0.214	29	5	Murakawa *et al.* (2011 ▶)
3ajx	1.60	828	*CNS*	0.004	1.2	0.004	1.3	0.198	0.218	8	0	Orita *et al.* (2010 ▶)
3ale	2.50	1460	*CNS*	0.007	1.3	0.004	1.3	0.227	0.279	117	15	Morita *et al.* (2010 ▶)
3am9	2.17	2584	*REFMAC*	0.022	1.9	0.008	6.9	0.182	0.248	99	12	Matsumoto *et al.* (2010 ▶)
3l9w	1.75	695	*REFMAC*	0.018	1.6	0.007	5.6	0.212	0.239	16	1	Roosild *et al.* (2010 ▶)
3lb4	1.56	274	*REFMAC*	0.011	1.3	0.005	4.2	0.253	0.281	10	0	Thompson *et al.* (2010 ▶)
3lfl	2.10	686	*PHENIX*	0.007	1.0	0.002	5.2	0.207	0.266	43	8	Zhou *et al.* (2011 ▶)
3lje	1.75	121	*CNS*	0.031	2.4	0.010	3.4	0.185	0.217	14	3	Pizzo *et al.* (2010 ▶)
3ljq	1.90	570	*CNS*	0.012	1.3	0.006	7.3	0.155	0.197	17	1	Wang Guo (2010 ▶)
3lju	1.70	373	*REFMAC*	0.016	1.4	0.007	6.0	0.203	0.238	1	0	Tong *et al.* (2010 ▶)
3lpf	2.26	1180	*REFMAC*	0.011	2.2	0.004	8.6	0.275	0.300	241	34	Wallace *et al.* (2010 ▶)
3lre	2.20	589	*REFMAC*	0.020	1.7	0.006	6.4	0.231	0.278	28	3	Peters *et al.* (2010 ▶)
3lrp	2.50	181	*CNS*	0.006	1.2	0.004	1.2	0.197	0.263	7	1	Cook *et al.* (2010 ▶)
3lt3	2.10	404	*REFMAC*	0.006	0.9	0.002	4.3	0.242	0.293	5	0	Biswas *et al.* (2010 ▶)
3m0e	2.63	1729	*PHENIX*	0.010	1.2	0.003	5.3	0.216	0.250	90	11	Chen, Sysoeva *et al.* (2010 ▶)
3m0h	1.58	1685	*CNS*	0.004	1.2	0.004	1.3	0.162	0.182	28	6	Yoshida *et al.* (2010 ▶)
3m4z	1.94	309	*REFMAC*	0.012	1.2	0.004	4.9	0.177	0.202	2	0	Heldman *et al.* (2010 ▶)
3m5o	1.60	410	*REFMAC*	0.009	1.2	0.004	5.9	0.240	0.270	10	3	Romano *et al.* (2010 ▶)
3m67	1.80	257	*REFMAC*	0.027	2.0	0.010	7.0	0.178	0.230	9	0	apkauskait *et al.* (2010 ▶)
3mbv	2.00	222	*REFMAC*	0.016	1.4	0.007	5.1	0.270	0.282	2	1	Borshchevskiy *et al.* (2010 ▶)
3mfa	1.63	194	*REFMAC*	0.013	1.3	0.004	6.9	0.198	0.223	8	0	Morin *et al.* (2011 ▶)
3mif	2.00	310	*REFMAC*	0.009	1.1	0.002	6.1	0.261	0.279	11	1	Chufn *et al.* (2010 ▶)
3mk9	2.08	173	*REFMAC*	0.006	1.1	0.005	1.0	0.216	0.242	7	0	Compton *et al.* (2011 ▶)
3mke	1.75	265	*REFMAC*	0.012	1.3	0.005	5.8	0.158	0.188	1	0	Ke *et al.* (2011 ▶)
3mvi	1.60	698	*REFMAC*	0.012	1.3	0.004	5.4	0.174	0.211	10	2	Niu *et al.* (2010 ▶)
3mxe	1.85	198	*REFMAC*	0.009	1.2	0.003	6.1	0.202	0.241	1	0	Ali *et al.* (2010 ▶)
3n2v	1.55	158	*REFMAC*	0.025	1.8	0.011	6.1	0.187	0.204	1	0	Attolino *et al.* (2010 ▶)
3nfy	1.94	498	*REFMAC*	0.019	1.8	0.007	6.3	0.187	0.279	23	6	Patterson *et al.* (2010 ▶)
3ni0	1.60	182	*PHENIX*	0.003	0.6	0.001	3.2	0.230	0.257	2	1	Swiecki *et al.* (2011 ▶)
3nk4	2.00	581	*PHENIX*	0.010	1.2	0.003	5.7	0.234	0.242	14	1	Han *et al.* (2010 ▶)
3nl6	2.61	1545	*PHENIX*	0.007	1.2	0.002	5.2	0.238	0.252	174	22	Paul *et al.* (2010 ▶)
3nm8	2.00	570	*REFMAC*	0.010	1.4	0.005	2.4	0.230	0.278	53	6	Sendovski *et al.* (2010 ▶)
3nmi	2.01	636	*REFMAC*	0.008	0.9	0.014	6.9	0.207	0.245	30	1	Radford *et al.* (2011 ▶)
3nof	1.60	213	*REFMAC*	0.029	2.5	0.011	5.9	0.197	0.225	19	3	Hall *et al.* (2011 ▶)
3nok	1.65	466	*REFMAC*	0.023	1.9	0.009	7.0	0.199	0.243	11	0	Carrillo *et al.* (2010 ▶)
3nv6	2.20	404	*REFMAC*	0.016	1.5	0.005	5.9	0.200	0.264	14	2	Yang *et al.* (2010 ▶)
3nxg	1.95	1291	*REFMAC*	0.008	1.1	0.002	5.9	0.180	0.213	21	0	Neu *et al.* (2010 ▶)
3nxp	2.20	363	*REFMAC*	0.011	1.3	0.003	6.1	0.213	0.239	11	2	Chen, Pelc *et al.* (2010 ▶)
3o0a	1.77	425	*REFMAC*	0.009	1.2	0.003	9.0	0.211	0.250	6	1	V. Cura, N. Olieric, E.-D. Wang, D. Moras, G. Eriani J. Cavarelli (unpublished work)
3o3p	1.70	635	*PHENIX*	0.007	1.1	0.002	5.2	0.221	0.255	39	4	Empadinhas *et al.* (2011 ▶)
3o4h	1.82	2302	*REFMAC*	0.019	1.7	0.007	6.2	0.244	0.267	71	19	Harmat *et al.* (2011 ▶)
3o79	1.60	202	*REFMAC*	0.022	1.7	0.009	5.3	0.210	0.237	4	2	Khan *et al.* (2010 ▶)
3o86	1.60	709	*PHENIX*	0.010	1.3	0.004	5.8	0.173	0.198	17	0	Eidam *et al.* (2010 ▶)
3oae	2.80	2105	*CNS*	0.011	1.5	0.006	1.4	0.272	0.288	238	40	Dasgupta *et al.* (2011 ▶)
3oag	2.30	669	*REFMAC*	0.009	1.2	0.003	7.8	0.208	0.262	13	2	Corminboeuf *et al.* (2010 ▶)
3oc2	1.97	495	*PHENIX*	0.007	1.1	0.002	5.4	0.181	0.223	21	3	Sainsbury *et al.* (2010 ▶)
3occ	1.70	1424	*REFMAC*	0.015	1.5	0.010	6.6	0.169	0.197	29	8	New York SGX Research Center for Structural Genomics (unpublished work)
3oep	1.75	485	*REFMAC*	0.006	1.1	0.003	5.4	0.191	0.215	0	0	Jacques *et al.* (2011 ▶)
3oi7	2.40	1028	*REFMAC*	0.012	1.3	0.003	5.9	0.237	0.269	19	8	Clasquin *et al.* (2011 ▶)
3oia	1.65	405	*REFMAC*	0.011	1.3	0.004	5.7	0.191	0.241	6	0	Lee *et al.* (2011 ▶)
3olz	2.75	743	*PHENIX*	0.006	0.9	0.002	5.0	0.211	0.265	26	1	Kumar Mayer (2010 ▶)
3om1	1.68	740	*PHENIX*	0.006	1.0	0.002	5.0	0.193	0.224	14	1	Kumar Mayer (2010 ▶)
3onw	2.38	702	*REFMAC*	0.009	1.1	0.002	5.1	0.251	0.280	13	1	Bosch *et al.* (2011 ▶)
3orv	1.91	1707	*REFMAC*	0.025	1.9	0.009	6.4	0.171	0.212	22	1	Abu Tarboush *et al.* (2010 ▶)
3oux	2.40	550	*PHENIX*	0.008	1.1	0.002	4.7	0.219	0.285	35	3	Sun Weis (2011 ▶)
3p10	1.70	471	*REFMAC*	0.012	1.3	0.004	5.2	0.184	0.218	7	0	Begley *et al.* (2011 ▶)
3p14	2.51	1608	*REFMAC*	0.019	1.8	0.005	6.5	0.201	0.266	108	22	T. T. N. Doan, P. Prabhu, J. K. Lee, L. W. Wang, J. K. Kim, M. Jeya Y. J. Ahn (unpublished work)
3p1a	1.70	281	*REFMAC*	0.015	1.5	0.006	5.1	0.169	0.211	4	0	Structural Genomics Consortium (unpublished work)
3p1m	2.54	1017	*REFMAC*	0.013	1.3	0.003	5.8	0.240	0.270	28	2	Structural Genomics Consortium (unpublished work)
3p2e	1.68	402	*CNS*	0.005	1.2	0.005	1.4	0.207	0.244	16	0	Husain *et al.* (2011 ▶)
3p32	1.90	306	*REFMAC*	0.015	1.3	0.004	5.6	0.226	0.254	3	0	Seattle Structural Genomics Center for Infectious Disease (unpublished work)
3p4i	2.35	760	*REFMAC*	0.016	1.5	0.003	5.7	0.200	0.244	11	0	Seattle Structural Genomics Center for Infectious Disease (unpublished work)
3p4l	1.80	198	*CNS*	0.010	1.6	0.008	2.2	0.205	0.240	7	2	Yang *et al.* (2011 ▶)
3p5o	1.60	127	*REFMAC*	0.007	1.0	0.003	4.9	0.179	0.207	1	0	Nicodeme *et al.* (2010 ▶)
3p5t	2.70	1653	*REFMAC*	0.010	1.3	0.003	6.1	0.225	0.272	179	33	Li, Tong *et al.* (2011 ▶)
3p77	1.60	371	*REFMAC*	0.012	1.4	0.005	6.1	0.194	0.226	2	0	Hee *et al.* (2010 ▶)
3p7h	2.30	520	*REFMAC*	0.110	1.2	0.004	6.5	0.204	0.245	16	2	Chatwell *et al.* (2008 ▶)
3p8s	2.00	302	*REFMAC*	0.010	1.2	0.003	6.2	0.192	0.226	3	0	U. Sharma, N. Ahmed, M. V. Krishnasastry C. G. Suresh (unpublished work)
3paj	2.00	599	*REFMAC*	0.009	1.2	0.007	2.0	0.213	0.259	20	2	Center for Structural Genomics of Infectious Diseases (unpublished work)
3pde	1.75	1149	*REFMAC*	0.013	1.6	0.004	5.0	0.177	0.209	17	0	New York SGX Research Center for Structural Genomics (unpublished work)
3pdt	1.80	251	*REFMAC*	0.011	1.3	0.003	5.9	0.202	0.247	4	0	Crawley *et al.* (2011 ▶)
3peh	2.75	523	*REFMAC*	0.013	1.7	0.007	2.5	0.236	0.279	8	1	Structural Genomics Consortium (unpublished work)
3pgj	2.49	1075	*REFMAC*	0.007	1.4	0.004	0.9	0.238	0.279	40	1	Center for Structural Genomics of Infectious Diseases (unpublished work)
3pgy	1.92	1609	*REFMAC*	0.017	1.5	0.006	5.7	0.190	0.227	29	2	Center for Structural Genomics of Infectious Diseases (unpublished work)
3ph7	2.50	1376	*BUSTER*	0.014	1.7	0.008	2.4	0.249	0.291	11	0	Artz *et al.* (2011 ▶)
3phe	2.20	2232	*CNX*	0.004	0.7	0.003	5.1	0.222	0.265	131	15	Kumar *et al.* (2011 ▶)
3pj9	2.10	549	*REFMAC*	0.017	1.5	0.005	6.2	0.206	0.221	16	2	Center for Structural Genomics of Infectious Diseases (unpublished work)
3pjp	1.60	389	*PHENIX*	0.006	1.0	0.003	5.6	0.214	0.251	5	0	Sun *et al.* (2010 ▶)
3pk0	1.74	1043	*REFMAC*	0.017	1.5	0.007	5.5	0.184	0.210	5	0	Seattle Structural Genomics Center for Infectious Disease (unpublished work)
Total		66891								2639	349	
Mean				0.014	1.4	0.005	5.1	0.207	0.243			
No. per 100 residues										4.0	0.5	

† As calculated in *PrimeX* without TLS scaling.

‡ A clash occurs when two atoms approach to within less than or equal to 0.8 times the sum of their van der Waals radii but greater than 0.7 times that sum.

§ A severe clash occurs when two atoms approach to within less than or equal to 0.7 times the sum of their van der Waals radii.

¶ L.Tresaugues, M. Welin, C. H. Arrowsmith, H. Berglund, C. Bountra, R. Collins, A. M. Edwards, S. Flodin, A. Flores, S. Graslund, M. Hammarstrom, I. Johansson, T. Karlberg, S. Kol, T. Kotenyova, E. Kouznetsova, M. Moche, T. Nyman, C. Persson, H. Schuler, P. Schutz, M. I. Siponen, A. G. Thorsell, S. Van der Berg, E. Wahlberg, J. Weigelt P. Nordlund (unpublished work).

**Table 4:** Statistics for structures in the moderate-resolution data set as refined in *PrimeX*

PDB code	Resolution ()	No. of residues	Bond-length r.m.s.d. ()	Bond-angle r.m.s.d. ()	Side-chain planarity r.m.s.d. ()	-Angle standard deviation ()	*R* factor	*R*_free_	Clashes	Severe clashes
2x3k	2.50	1145	0.019	2.3	0.005	8.0	0.212	0.267	19	0
2xda	1.85	150	0.025	2.5	0.007	6.9	0.175	0.215	3	0
2xn8	1.64	409	0.022	2.2	0.005	6.6	0.170	0.221	2	0
2xpp	1.74	161	0.016	1.9	0.005	5.2	0.197	0.251	3	0
2xs6	2.09	166	0.019	2.0	0.004	6.8	0.195	0.263	1	0
2xsn	2.68	1341	0.017	2.0	0.005	6.6	0.229	0.275	16	0
2xsq	1.72	179	0.022	2.1	0.009	6.4	0.158	0.195	0	0
2xsw	1.90	666	0.021	2.3	0.007	7.0	0.166	0.211	5	0
2xsx	1.70	869	0.023	2.1	0.007	6.4	0.162	0.203	4	0
2xu7	1.90	752	0.020	2.2	0.006	8.0	0.186	0.235	1	0
2xul	2.20	645	0.016	2.2	0.003	7.2	0.189	0.231	1	0
2xvs	1.80	166	0.022	2.3	0.008	6.9	0.177	0.252	1	0
2xvv	2.40	582	0.019	2.3	0.005	6.8	0.182	0.245	3	0
2xxj	1.96	1239	0.017	1.9	0.007	6.5	0.179	0.241	8	0
3acw	1.63	284	0.019	2.0	0.009	5.8	0.201	0.242	1	0
3aey	1.92	698	0.021	2.2	0.008	6.3	0.171	0.204	12	0
3ajx	1.60	828	0.022	2.2	0.006	5.7	0.178	0.211	1	0
3ale	2.50	1460	0.015	2.1	0.004	7.5	0.233	0.272	16	0
3am9	2.17	2584	0.017	2.1	0.005	7.1	0.175	0.241	23	0
3l9w	1.75	695	0.023	2.4	0.009	6.5	0.199	0.233	3	0
3lb4	1.56	274	0.018	2.3	0.012	5.1	0.244	0.277	1	0
3lfl	2.10	686	0.017	1.9	0.003	8.5	0.202	0.269	8	0
3lje	1.75	121	0.025	2.4	0.006	6.5	0.175	0.211	1	0
3ljq	1.90	570	0.020	2.2	0.006	7.2	0.144	0.200	5	0
3lju	1.70	373	0.021	2.2	0.007	6.9	0.192	0.237	1	0
3lpf	2.26	1180	0.017	2.3	0.005	10.2	0.280	0.325	30	1
3lre	2.20	589	0.016	2.1	0.005	7.0	0.218	0.273	2	0
3lrp	2.50	181	0.017	2.2	0.004	7.1	0.184	0.252	3	0
3lt3	2.10	404	0.019	2.4	0.005	7.5	0.188	0.282	2	0
3m0e	2.63	1729	0.015	1.9	0.004	6.8	0.236	0.273	18	0
3m0h	1.58	1685	0.022	2.2	0.008	5.8	0.144	0.178	9	0
3m4z	1.94	309	0.020	2.1	0.007	6.1	0.160	0.203	0	0
3m5o	1.60	410	0.015	2.0	0.007	7.0	0.206	0.247	2	2
3m67	1.80	257	0.026	2.6	0.005	7.9	0.168	0.226	4	0
3mbv	2.00	222	0.017	1.8	0.006	5.9	0.248	0.292	1	0
3mfa	1.63	194	0.021	2.3	0.012	8.6	0.181	0.225	1	0
3mif	2.00	310	0.021	2.4	0.008	9.3	0.194	0.242	3	0
3mk9	2.08	173	0.017	1.9	0.006	6.8	0.186	0.246	2	0
3mke	1.75	265	0.020	2.1	0.010	6.8	0.148	0.191	0	0
3mvi	1.60	698	0.023	2.2	0.008	6.3	0.162	0.205	3	0
3mxe	1.85	198	0.019	2.3	0.005	7.7	0.183	0.251	0	0
3n2v	1.55	158	0.023	2.4	0.008	7.2	0.178	0.227	0	0
3nfy	1.94	498	0.017	2.1	0.004	6.9	0.181	0.259	6	0
3ni0	1.60	182	0.017	1.9	0.003	5.2	0.213	0.254	0	0
3nk4	2.00	581	0.021	2.5	0.006	7.8	0.217	0.247	7	0
3nl6	2.61	1545	0.016	2.4	0.005	7.8	0.225	0.273	23	2
3nm8	2.00	570	0.018	2.2	0.007	7.4	0.191	0.262	2	0
3nmi	2.01	636	0.023	2.4	0.005	6.8	0.198	0.244	9	1
3nof	1.60	213	0.021	2.4	0.010	6.5	0.186	0.216	2	0
3nok	1.65	466	0.016	2.0	0.005	7.4	0.198	0.243	4	0
3nv6	2.20	404	0.015	2.0	0.004	6.6	0.196	0.260	0	0
3nxg	1.95	1291	0.018	2.2	0.006	7.5	0.166	0.213	8	0
3nxp	2.20	363	0.015	2.1	0.005	8.1	0.196	0.238	9	0
3o0a	1.77	425	0.019	2.2	0.007	7.2	0.187	0.248	2	2
3o3p	1.70	635	0.020	2.5	0.006	8.2	0.216	0.251	14	0
3o4h	1.82	2302	0.015	2.2	0.005	6.5	0.216	0.270	16	2
3o79	1.60	202	0.024	2.5	0.012	11.1	0.192	0.239	2	1
3o86	1.60	709	0.021	2.1	0.007	6.8	0.167	0.196	4	0
3oae	2.80	2105	0.017	2.5	0.005	9.4	0.237	0.294	39	0
3oag	2.30	669	0.015	2.1	0.005	8.3	0.210	0.261	1	0
3oc2	1.97	495	0.019	2.3	0.007	7.5	0.171	0.226	5	1
3occ	1.70	1424	0.021	2.1	0.007	7.1	0.156	0.192	15	5
3oep	1.75	485	0.020	2.2	0.008	6.5	0.168	0.210	4	0
3oi7	2.40	1028	0.015	1.9	0.005	6.9	0.241	0.280	7	0
3oia	1.65	405	0.022	2.3	0.008	7.0	0.171	0.237	5	0
3olz	2.75	743	0.014	2.0	0.003	6.8	0.238	0.264	10	0
3om1	1.68	740	0.022	2.4	0.007	6.9	0.183	0.223	6	0
3onw	2.38	702	0.018	2.2	0.005	7.4	0.225	0.263	6	0
3orv	1.91	1707	0.017	2.0	0.006	7.2	0.155	0.207	10	0
3oux	2.40	550	0.017	2.2	0.004	6.4	0.220	0.267	8	0
3p10	1.70	471	0.018	2.1	0.006	6.3	0.173	0.222	4	0
3p14	2.51	1608	0.015	2.1	0.005	7.1	0.211	0.259	21	5
3p1a	1.70	281	0.023	2.0	0.009	5.8	0.162	0.212	2	0
3p1m	2.54	1017	0.013	2.1	0.002	6.9	0.231	0.273	8	0
3p2e	1.68	402	0.021	2.2	0.008	6.6	0.186	0.234	3	0
3p32	1.90	306	0.024	2.3	0.005	7.3	0.204	0.252	3	0
3p4i	2.35	760	0.019	2.1	0.004	7.0	0.195	0.241	2	0
3p4l	1.80	198	0.018	2.0	0.008	7.2	0.200	0.248	1	0
3p5o	1.60	127	0.023	2.1	0.008	6.1	0.156	0.209	1	0
3p5t	2.70	1653	0.016	2.0	0.004	7.4	0.230	0.285	4	0
3p77	1.60	371	0.021	2.3	0.008	6.8	0.173	0.221	2	0
3p7h	2.30	520	0.016	2.0	0.005	8.1	0.200	0.244	2	0
3p8s	2.00	302	0.018	2.2	0.006	7.7	0.165	0.221	2	0
3paj	2.00	599	0.018	2.2	0.006	6.2	0.179	0.251	6	0
3pde	1.75	1149	0.020	2.1	0.006	6.1	0.169	0.212	8	0
3pdt	1.80	251	0.018	2.0	0.005	7.6	0.182	0.237	2	0
3peh	2.75	523	0.019	2.6	0.006	8.4	0.234	0.288	10	0
3pgj	2.49	1075	0.014	1.9	0.003	7.0	0.249	0.287	3	0
3pgy	1.92	1609	0.017	2.0	0.005	6.7	0.173	0.225	8	0
3ph7	2.50	1376	0.016	2.0	0.006	6.4	0.248	0.304	8	0
3phe	2.20	2232	0.016	2.0	0.004	7.7	0.213	0.255	11	1
3pj9	2.10	549	0.021	2.2	0.004	7.3	0.185	0.220	10	0
3pjp	1.60	389	0.022	2.4	0.008	7.7	0.203	0.253	2	0
3pk0	1.74	1043	0.023	2.3	0.007	6.2	0.160	0.207	7	0
Total		66891							573	23
Mean	1.99		0.019	2.2	0.006	7.1	0.193	0.242		
No. per 100 residues									0.9	0.03

**Table 5:** Summary of geometry and clash statistics

Structure set	No. of structures	Bond-length r.m.s.d. ()	Bond-angle r.m.s.d. ()	Side-chain planarity r.m.s.d. ()	-Angle standard deviation ()	Clashes per 100 residues	Severe clashes per 100 residues
Ultrahigh-resolution set	18	0.021	2.5	0.011	6.4	0.6†	0.03†
Moderate-resolution set + additional *PrimeX* refinement	94	0.019	2.2	0.006	7.1	0.7‡	0.03‡
Moderate-resolution set as deposited	94	0.015	1.4	0.005	5.1	4.0	0.5
*BUSTER*-refined subset	2	0.014	1.7	0.008	2.6	0.9	0.07
*CNS*/*CNX*-refined subset	12	0.009	1.3	0.005	2.3	5.9	0.8
*PHENIX*-refined subset	14	0.008	1.1	0.003	5.4	4.7	0.5
*REFMAC*-refined subset	66	0.016	1.5	0.005	5.7	3.4	0.5

† Corrected for obvious errors in deposited structures, as shown in Tables 1 ▶ and 2 ▶.

‡ Corrected for clashes owing to errors in the structures (see text); the numbers of uncorrected clashes and severe clashes per 100 residues are 0.9 and 0.03, respectively.

## Footnotes

1 Supplementary material has been deposited in the IUCr electronic archive (Reference: RR5017). Services for accessing this material are described at the back of the journal.
