# Supplementary material for: Significant reduction in errors associated with nonbonded contacts in protein crystal structures: automated all-atom refinement with PrimeX
Source: Acta Crystallogr D Biol Crystallogr. 2012 Jul 17;68(Pt 8):935–52. doi: 10.1107/S0907444912017453 (PMC3413210; doi:10.1107/S0907444912017453)
Supplement: Supplementary file 1 [file d-68-00935-sup1.pdf]

Significant reduction in errors associated with non-bonded contacts in protein crystal structures: automated all-atom refinement with *PrimeX*

Jeffrey A. Bell, Kenneth L. Ho and Ramy Farid

Supplementary Material

Table S1. Ramachandran Z-score, bond length RMS Z-scores, and bond angle RMS Z-scores, as calculated in WHAT_CHECK (Hooft *et al*., 1996b) for each protein as deposited and after refinement with the polish workflow in PrimeX.

PDB	Ramachandran Z-score		Bond length RMS Z-score		Bond angle RMS Z-score	
ID	As deposited	PrimeX-refined	As deposited	PrimeX-refined	As deposited	PrimeX-refined
2X3K	-2.32	-1.99	0.68	0.94	0.80	1.30
2XDA	0.96	-0.06	0.65	1.16	0.68	1.36
2XN8	-0.48	-0.63	0.99	1.02	0.92	1.21
2XPP	1.21	0.74	0.77	0.72	0.79	1.02
2XS6	0.08	-0.38	0.36	0.76	0.55	1.07
2XSN	-0.01	-1.13	0.62	0.82	0.89	1.08
2XSQ	0.13	0.01	0.43	1.03	0.67	1.12
2XSW	0.50	0.21	0.48	0.99	0.67	1.23
2XSX	-0.13	-0.35	0.67	1.05	0.74	1.14
2XU7	-0.88	-0.61	0.98	0.95	0.94	1.18
2XUL	-1.24	-0.82	0.87	0.76	0.93	1.11
2XVS	-0.03	-0.40	0.69	1.01	0.77	1.21
2XVV	-2.36	-1.93	0.31	0.88	0.61	1.25
2XXJ	-0.70	-1.29	0.32	0.77	0.55	1.03
3ACW	1.56	0.88	0.32	0.90	0.79	1.08
3AEY	-0.99	-0.86	0.25	0.97	0.59	1.14
3AJX	0.10	0.01	0.25	1.02	0.56	1.49
3ALE	-2.50	-1.48	0.32	0.72	0.66	1.32
3AM9	-0.90	-0.50	0.96	0.68	0.95	1.03
3L9W	0.53	0.20	0.78	1.11	0.86	1.30
3LB4	1.40	0.86	0.53	0.72	0.69	1.23
3LFL	-0.58	0.06	0.33	0.78	0.50	1.07
3LJE	-0.20	-0.19	1.24	1.12	1.19	1.30
3LJQ	0.37	0.31	0.55	0.94	0.68	1.18
3LJU	0.28	-0.25	0.69	1.02	0.75	1.20
3LPF	-2.64	-2.44	0.53	0.83	1.00	1.26
3LRE	-0.42	0.08	0.77	0.79	0.88	1.10
3LRP	-1.91	-1.27	0.30	0.82	0.61	1.21
3LT3	-1.17	-1.79	0.37	0.89	0.48	1.27
3M0E	-1.10	-1.02	0.41	0.67	0.61	1.02
3M0H	0.18	-0.12	0.23	1.01	0.59	1.20
3M4Z	0.40	-0.10	0.54	0.97	0.65	1.12
3M5O	0.54	0.09	0.41	0.71	0.66	1.04
3M67	-1.56	-1.58	1.18	1.24	1.02	1.41
3MBV	-0.10	-0.66	0.72	0.81	0.70	1.05
3MFA	-0.18	-0.07	0.62	1.03	0.65	1.21
3MIF	-0.30	-0.75	0.45	1.01	0.61	1.30
3MK9	0.53	0.87	0.29	0.79	0.54	1.03
3MKE	0.38	0.18	0.53	0.85	0.66	1.07
3MVI	-0.03	-0.26	0.53	1.07	0.68	1.19
3MXE	-0.15	-0.52	0.43	0.87	0.59	1.15

3N2V	0.25	0.00	1.10	0.91	0.96	1.15
3NFY	-0.48	-0.08	0.87	0.78	0.91	1.12
3NI0	3.69	3.05	0.15	0.94	0.37	1.10
3NK4	-0.09	-0.12	0.43	1.03	0.67	1.34
3NL6	-3.16	-2.11	0.34	0.65	0.59	1.22
3NM8	-1.08	-0.91	0.45	0.88	0.70	1.24
3NMI	0.22	0.09	0.37	1.07	0.47	1.33
3NOF	-0.12	0.28	1.27	0.97	1.25	1.30
3NOK	-0.32	-0.19	1.02	0.74	0.97	1.00
3NV6	-1.52	-1.19	0.71	0.74	0.79	1.09
3NXG	-0.18	-0.34	0.38	0.82	0.58	1.15
3NXP	-1.20	-1.08	0.51	0.71	0.66	1.11
3O0A	0.18	-0.44	0.42	0.90	0.59	1.15
3O3P	-1.84	-2.56	0.32	0.96	0.57	1.31
3O4H	-0.25	-0.86	0.82	0.72	0.86	1.17
3O79	1.38	1.19	0.98	1.11	0.93	1.34
3O86	0.07	-0.19	0.45	0.97	0.63	1.12
3OAE	-2.87	-2.32	0.48	0.80	0.70	1.35
3OAG	-1.86	-1.76	0.43	0.71	0.62	1.12
3OC2	-0.48	-0.82	0.32	0.88	0.57	1.24
3OCC	-0.59	-0.76	0.66	0.97	0.74	1.11
3OEP	-0.05	-0.44	0.30	0.90	0.58	1.16
3OI7	-0.60	-0.37	0.57	0.72	0.70	1.03
3OIA	-1.00	-1.21	0.49	1.04	0.70	1.26
3OLZ	-1.51	-0.35	0.31	0.72	0.56	1.08
3OM1	0.74	0.26	0.31	0.99	0.56	1.27
3ONW	-0.78	-1.84	0.42	0.84	0.56	1.18
3ORV	-0.77	-0.78	1.05	0.83	0.95	1.10
3OUX	-2.13	-1.68	0.35	0.81	0.59	1.17
3P10	-0.17	-0.50	0.58	0.87	0.70	1.13
3P14	-1.57	-0.92	0.81	0.76	0.92	1.13
3P1A	0.84	0.62	0.64	1.05	0.79	1.08
3P1M	-0.17	-0.38	0.59	0.66	0.70	1.10
3P2E	-0.33	-0.48	0.25	0.99	0.55	1.22
3P32	0.31	-0.68	0.69	1.14	0.69	1.24
3P4I	-0.52	-0.62	0.74	0.97	0.77	1.14
3P4L	-0.51	-0.29	0.45	0.85	0.75	1.07
3P5O	0.99	0.21	0.33	1.01	0.47	1.14
3P5T	-1.80	-0.80	0.44	0.77	0.67	1.08
3P77	1.33	0.74	0.52	1.00	0.71	1.22
3P7H	-1.18	-0.84	0.50	0.76	0.62	1.10
3P8S	-1.24	-1.31	0.42	0.81	0.54	1.13
3PAJ	1.06	0.30	0.57	0.86	0.80	1.17
3PDE	0.42	-0.38	0.51	0.96	0.65	1.13
3PDT	0.45	0.13	0.45	0.82	0.60	1.09
3PEH	-0.95	-2.09	0.64	0.91	0.91	1.36
3PGJ	-1.31	-1.47	0.33	0.66	0.69	1.07
3PGY	-0.14	-0.56	0.74	0.86	0.77	1.08
3PH7	-0.20	-1.47	0.67	0.76	0.90	1.09
3PHE	-0.52	-0.83	0.20	0.79	0.44	1.09
3PJ9	-0.14	-0.22	0.72	0.95	0.73	1.17
3PJP	0.68	0.13	0.27	1.01	0.54	1.26
3PK0	0.34	-0.25	0.74	1.06	0.77	1.18

Table S2. Bond length and bond angle RMS Z-scores, as calculated in WHAT_CHECK (Hooft *et al.*, 1996b) for each protein in the ultra-high-resolution data set.

PDB ID	Bond length RMS Z-scores	Bond angle RMS Z-score
1BYZ	0.79	0.87
1DY5	0.91	1.29
1I1W	1.13	1.26
1M40	0.69	1.24
1MUW	0.82	1.23
1P9G	0.76	1.10
1UCS	0.64	1.07
1VYR	0.84	1.14
1YK4	0.99	1.13
2B97	1.23	1.39
2H5C	0.74	1.00
2VB1	0.92	1.18
2WUR	1.48	1.47
2XU3	0.49	0.73
3A38	0.90	1.14
3G63	0.71	1.05
3IP0	0.53	0.79
3MI4	1.07	1.13
